# Resonance Raman Optical Activity Spectroscopy in Probing Structural Changes Invisible to Circular Dichroism Spectroscopy: A Study on Truncated Vitamin B_12_ Derivatives

**DOI:** 10.3390/molecules25194386

**Published:** 2020-09-24

**Authors:** Ewa Machalska, Grzegorz Zajac, Monika Halat, Aleksandra J. Wierzba, Dorota Gryko, Malgorzata Baranska

**Affiliations:** 1 Faculty of Chemistry, Jagiellonian University, Gronostajowa 2, 30-387 Krakow, Poland; ewa.machalska@doctoral.uj.edu.pl (E.M.); monika.halat@doctoral.uj.edu.pl (M.H.); 2 Jagiellonian Centre for Experimental Therapeutics (JCET), Jagiellonian University, Bobrzynskiego 14, 30-348 Krakow, Poland; zajac@chemia.uj.edu.pl; 3 Institute of Organic Chemistry, Polish Academy of Sciences, Kasprzaka 44/52, 01-224 Warsaw, Poland; aleksandra.wierzba@icho.edu.pl

**Keywords:** vitamin B_12_, cobinamide, chirality, resonance ROA, resonance Raman spectroscopy, ECD

## Abstract

This work demonstrates resonance Raman optical activity (RROA) spectra of three truncated vitamin B_12_ derivatives modified within the nucleotide loop. Since truncated cobalamins possess sufficiently high rotational strength in the range of ROA excitation (532 nm), it was possible to record their spectra in the resonance condition. They showed several distinct spectral features allowing for the distinguishing of studied compounds, in contrast to other methods, i.e., UV-Vis absorption, electronic circular dichroism, and resonance Raman spectroscopy. The improved capacity of the RROA method is based here on the excitation of molecules via more than two electronic states, giving rise to the bisignate RROA spectrum, significantly distinct from a parent Raman spectrum. This observation is an important step in the dissemination of using RROA spectroscopy in studying the complex structure of corrinoids which may prove crucial for a better understanding of their biological role.

## 1. Introduction

**Figure 1:**
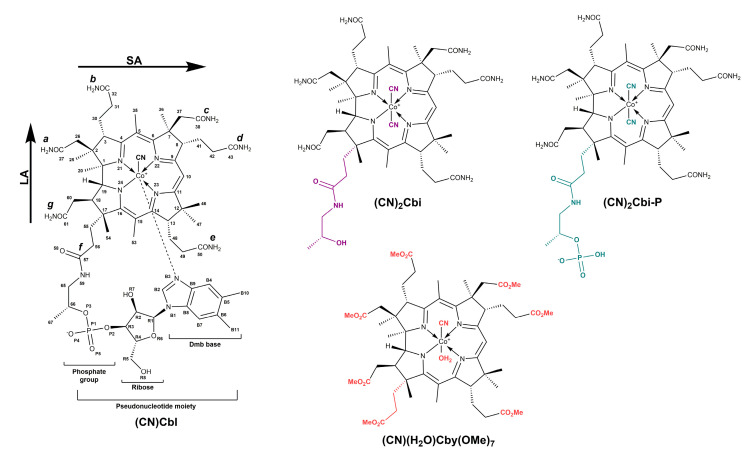
Molecular structure of vitamin B_12_ and its truncated analogs: dicyanocobinamide ((CN)_2_Cbi) and its phosphate derivative ((CN)_2_Cbi-P), and cyanoaqua cobyrinic acid heptamethylester ((CN)(H_2_O)Cby(OMe)_7_).

Natural cobalt-corrinoids and their synthetic analogs have recently been widely explored as transporters of biologically important molecules into eukaryotic or prokaryotic cells [1,2], fluorophore probes [3], antivitamins [4,5], and a catalyst [6] like other commonly known catalytic materials [7]. Vitamin B_12_ (cyanocobalamin, (CN)Cbl), belonging to the best studied among all corrinoid molecules, is a micronutrient isolated from specific bacterial sources e.g., *Propionibacteria* and *Pseudomonas denitnpcans* [8,9]. This coordination complex (Figure 1) comprises several relevant structural entities: (1) chiral tetrapyrrolic corrin ring with the central cobalt ion in +3 oxidation state; (2) the nucleotide loop composed of the phosphate group, ribose unit, and 5,6-dimethylbenzimidazole base (Dmb), coordinated to the cobalt atom in the lower axial position (L_α_); (3) CN^−^ group as an upper ligand (L_β_). Vitamin B_12_ seems to have no physiological function in human metabolism [10], nevertheless, organometallic cobalamin cofactors: adenosylcobalamin (AdoCbl) and methylcobalamin (MeCbl) show an enzymatic capacity [11,12]. AdoCbl is used for initiating the radical-induced rearrangement of substrates [13] or the reduction of ribonucleotides [14], while MeCbl participates in the transfer of the methyl cation [15].

Important representatives of the corrinoid family are truncated vitamin B_12_ derivatives such as cobyric acid (Cby) and cobinamide (Cbi), obtained via hydrolysis of native cobalamin (Figure 1) [16,17]. The molecular structure of both compounds, Cby and Cbi, is not as complex as the one of vitamin B_12_, due to modifications within the *a*-*f* side chains or cleavage of the phosphodiester linkage, respectively [18]. The absence of the Dmb base in the α-position of these species not only contributes to richer coordination possibilities, but ensures that aqua Cbi is a more sensitive probe for cyanide poisoning and its detection, than other generally known antidotes, including sodium nitrite, sodium thiosulfate, and hydroxocobalamin (OHCbl) [19,20]. It was noted that Cbi species bind cyanide with high affinity (*K_a_* ≈ 10^22^ M^−1^), which leads to 2–3 times greater detoxification efficiency, compared to that of cobalamins [21]. The role of Cbi is not restricted to cyanide neutralization, it is also used to study the function of the lower axial ligand in base-off/His-on cobalamin-dependent enzymes e.g., methylmalonyl-CoA mutase [22].

Until now, vitamin B_12_ and its derivatives have been extensively investigated by means of various methods including electronic absorption (UV-Vis) [23,24,25,26] as well as Raman spectroscopy techniques [27,28,29,30,31,32]. These methods supported by a theoretical approach guarantee insight into the complex nature and electronic or vibrational properties of corrinoids. As it turned out, there is a range of factors influencing the electronic character of the corrin chromophore e.g., (1) type of the central metal ion [33,34]; (2) type of axial ligands attached to the metal ion [35,36,37]; (3) oxidation state of the cobalt center [27,38]; (4) character of substituents attached to the corrin ring or presence/absence of the nucleotide loop [18,39]. Also, electronic circular dichroism spectroscopy (ECD) has proven to be a sensitive method for the detection of a substitution at the central cobalt ion or functionalization of the corrinoid ring [37,40]. However, given the character of the ECD method, arising from the presence of a few intense, but broad, signatures in the chiroptical spectra, this technique yields limited information about the molecular structure.

We have previously shown that for variously modified cobalamin species, resonance Raman optical activity (RROA) spectroscopy is a method of enhanced structural sensitivity compared to other techniques, including resonance Raman (RR) spectroscopy, and at the same time preserves improved sensitivity as related to the ROA effect itself [41]. According to the single electronic state (SES) limit theory, the RROA spectrum is monosignate with a sign opposite to the ECD signal of the single resonant electronic state. Furthermore, the RROA/RR CID ratio of corresponding bands equals −*g*/2, where the *g*-factor or dissymmetry factor is the ECD/UV-Vis ratio of the resonant electronic state. Excitation via a single electronic state results in the high similarity of an RROA spectrum to an RR spectrum (corresponding signals have the same intensities, apart from the sign) [42]. On the other hand, the excitation via more than one electronic state, described by two electronic states (TES) [43] or multiple electronic states [44] limit theory, may give rise to a bisignate RROA spectrum. This effect is significantly distinct from the Raman spectrum of a studied molecule and does not obey the CID = −*g*/2 rule. The bisignated RROA spectrum may also result from conformational freedom of the molecule [45,46], or weak enhancement, and the presence of non-resonance bands together with the resonantly increased ones [47], and resonance with more than two electronic states [44]. In respect to vitamin B_12_, the bisignate RROA spectra result from the excitation (532 nm) involving at least three closely-lying electronic states, which agrees with the theoretical analysis of their ECD spectra [40].

In this work, we demonstrate the experimental RROA spectra for three vitamin B_12_ mimics, with a modified nucleotide loop: dicyanocobinamide ((CN)_2_Cbi) and its phosphate analog ((CN)_2_Cbi-P) as well as cyanoaqua cobyrinic acid heptamethylester ((CN)(H_2_O)Cby(OMe)_7_) (Figure 1). Due to the fact that corrinoid molecules possess sufficiently high rotational strength in the range of ROA excitation (532 nm), it was possible to record their RROA spectra. They showed several distinct spectral features enabling the differentiation of the studied compounds, in contrast to other methods, like UV-Vis, ECD, and resonance Raman spectroscopy.

## 2. Results and Discussion

**Figure 2:**
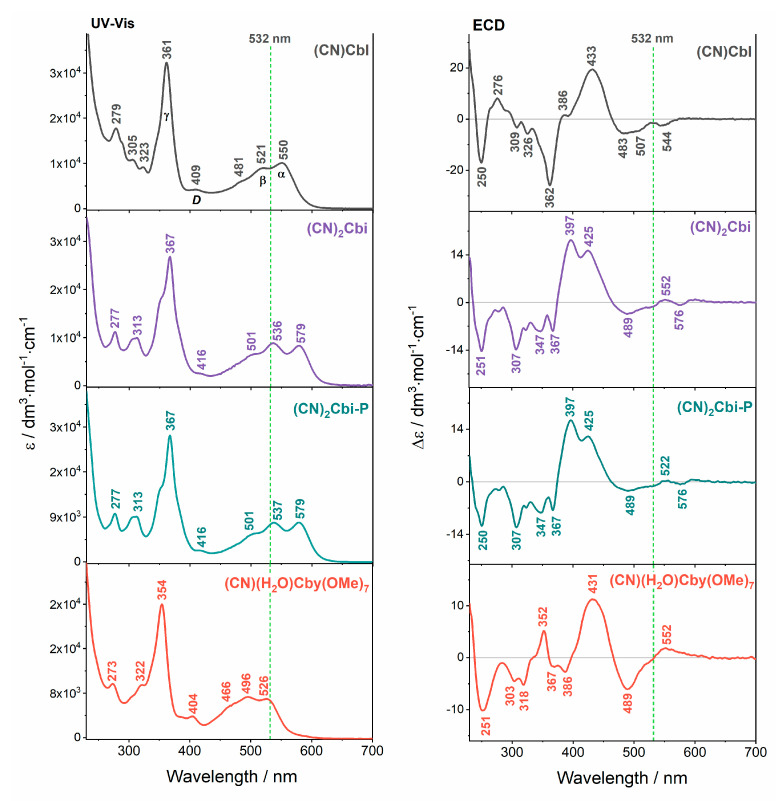
Experimental UV-Vis and ECD spectra of vitamin B_12_ and its truncated analogs. The green dotted line indicates the excitation wavelength of the ROA laser.

The UV-Vis and ECD spectra of vitamin B_12_ in an aqueous solution are presented, along with spectra of its truncated derivatives in Figure 2 and Figure S1 (Supplementary Materials). The absorption spectra of (CN)Cbl [40] and some of its incomplete analogs in organic and non-organic solvents [18,37,48,49] have been previously reported and they agree well with the spectra obtained in this study. The spectral profiles and electronic properties of corrinoid molecules slightly change depending on the solvent used [36,48]. As generally proposed and subsequently confirmed, the UV-Vis spectra are mostly dominated by intense corrin-based π→π* transitions, since (CN)Cbl and its derivatives closely resemble those possessing metal-free corrin rings [40,50].

It is also worth emphasizing that the UV-Vis spectra of (CN)Cbl and its truncated analogs presented several similarities: the main absorption bands α/β, *D*, and γ appeared in the spectra of all studied species and are of comparable intensities (Figure 2). The manifestation of these unique absorption features leads to the classification of the studied corrinoids’ UV-Vis spectra to the so-called *normal* [37,51]. Nevertheless, based on the changes in the color of the (CN)Cbl solution in water compared to its incomplete derivatives: from red for cobalamin to rosy for (CN)(H_2_O)Cby(OMe)_7_ and purple for (CN)_2_Cbi and (CN)_2_Cbi-P, the absorption spectra of these compounds differs with regard to the position (wavelength) of distinctive bands. In the case of native (CN)Cbl, two prominent bands occurred in the α/β visible region with the more intense counterpart at 550 nm and less intense at 521 nm being the electronic origin and vibrational progression, respectively, of the electronic excitation from the orbital HOMO to the orbital LUMO of the corrin macrocycle. The absorption band of the highest intensity in the near-UV spectral region was centered at 361 nm and it was assigned to a transition with predominant corrin π→π* nature [27,40]. The “γ-band-type” transition results are generally from mixing between Co 3d and corrin-based π orbitals that produce destabilization of all 3d orbitals [24,40].

On the other hand, replacing the bulky Dmb base with CN^−^ or an H_2_O ligand perturbs the electronic structure of corrinoids. In the context of truncated vitamins, a few spectral features can be pointed out: (1) the α-band has comparable intensity to that of the β ones; (2) changing the lower axial ligand by CN^−^ results in red-shift of the α and β bands, except for cyanoaqua cobyrinic acid derivative; (3) the low-intensity band referred to as *D*, which was present in the *normal* spectra of both cobalamin and truncated vitamins, shifts towards higher or lower wavelengths compared to the (CN)Cbl (409 nm), respectively for (CN)_2_Cbi and its phosphate analog (416 nm) and (CN)(H_2_O)Cby(OMe)_7_ (404 nm); (4) a more pronounced shoulder on the higher energy side of the γ band was observed upon replacing the axial Dmb moiety with a different group. It is evident that the energies of α, β, and γ bands vary depending on the pair of axial substituents attached to the cobalt ion, in other words, the blue-shift of dominant bands can be noticed with increasing fashion in the donation of electrons by axial ligands ([CN^−^, CN^−^] < [CN^−^, H_2_O]). The blue-shifted absorption in the spectrum of (CN)(H_2_O)Cby(OMe)_7_ relative to that of (CN)Cbl can be attributed to the elimination of σ-antibonding interactions, producing a species-dependent stabilization of the HOMO compared to the LUMO orbital [24].

The experimental ECD spectra presented in Figure 2 show enhanced sensitivity over UV-Vis spectroscopy in probing slight changes in the electronic structure of cobalamin and its truncated derivatives, which are a consequence of an exchange of an axial ligand incorporation to the central cobalt ion. The ECD spectra of all studied corrinoids were dominated by an intense and broad positive signal between 400 and 500 nm and a series of negative bands in the range of 240 to 370 nm (the spectrum of (CN)(H_2_O)Cby(OMe)_7_ with a positive band at 352 nm is an exception). The spectral signatures are different e.g., (CN)Cbl vs. incomplete analogs. Presumably, the unique variances between chiroptical ECD spectra of the parent vitamin and its derivatives are a consequence of the replacement of the bulky Dmb base (a moderately strong σ-donor) with significantly smaller CN^−^ (moderate σ- and π-donor) or H_2_O ligand (rather weakly σ- and π-donor) [40], which leads to a greater deformation of the corrin ring. Interestingly, the UV-Vis and ECD spectra of two cobinamide species ((CN)_2_Cbi and (CN)_2_Cbi-P) were identical, superimposable curves. This proves that both spectroscopic methods (UV-Vis and ECD) were not sufficiently sensitive to subtle changes in the structure of truncated cobinamide bearing a free phosphate group vs. its native form. The minor changes in the absorption intensities and rotational strength of (CN)_2_Cbi and (CN)_2_Cbi-P species can be exclusively detected.

RR spectra of cobalamins and cobinamides were strongly dominated by the corrin-based vibrational modes, with the most intense band centered at ~1501 cm^−1^ associated with the long-axis polarized corrin macrocycle mode, *ν*_LA_, which was mainly attributed to the in-phase C=C and C=N stretching along the longer (C5-Co-C15) corrin ring axis (Figure 1). Another equally important spectral feature, close to the *ν*_LA_, was the short-axis polarized corrin ring motion: *ν*_SA_, located between 1543 and 1549 cm^−1^ and correlated with the C=C and C=N stretching along the shorter (Co-C10) corrin ring axis, and two other corrin macrocycle C=C stretching modes located above 1560 cm^−1^ [30]. The spectral region between 1300 and 1400 cm^−1^ can be generally assigned to the CH_3_ and CH_2_ bending vibrations, while the 1100 to 1300 cm^−1^ region to the CH_2_ twisting and wagging, CH bending, as well as the corrin ligand C-C and C-N stretching motions [41]. Moreover, the low wavenumber region below 550 cm^−1^ was more sensitive to the replacement of an axial ligand attached to the cobalt ion, and it was dominated by the Co-C stretching vibrations located around 500 cm^−1^, that was more intense for an alkyl substituted Co, as well as Co-C≡N bending or C≡N twisting modes, present for the analogs with the CN^−^ axial ligand (Table 1) [30,41].

**Figure 3:**
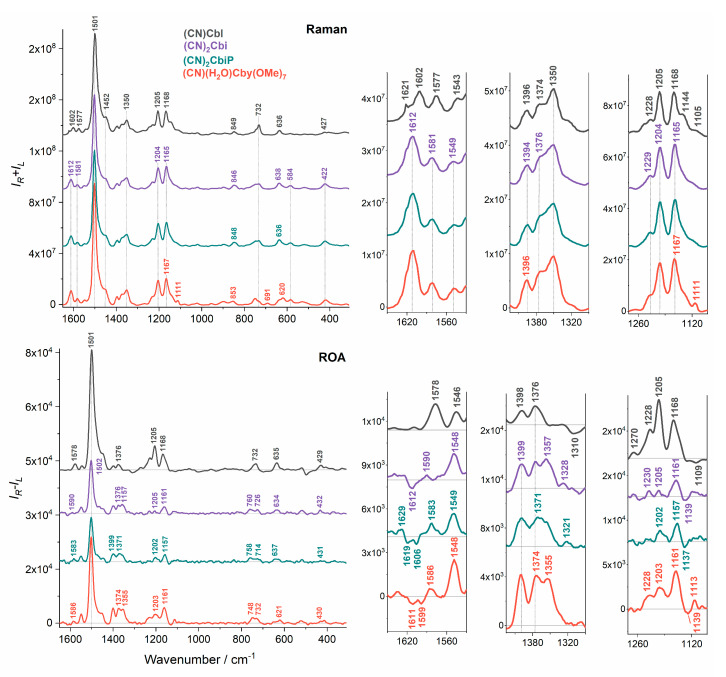
Experimental Raman and RROA spectra of vitamin B_12_ and its truncated analogs.

**Figure 4:**
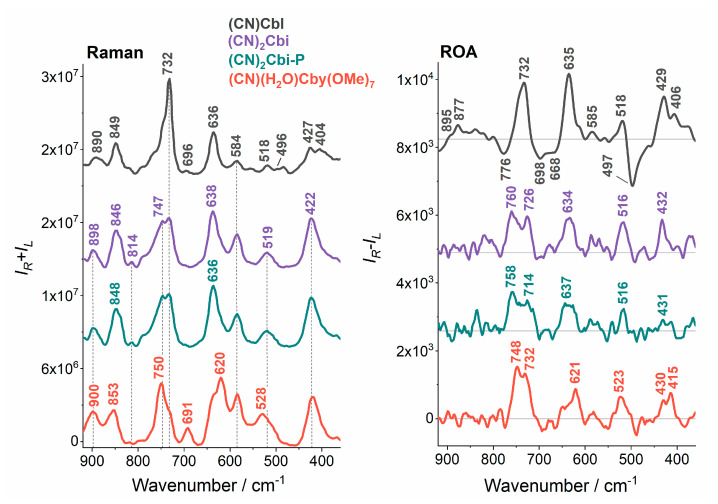
Detailed comparison of Raman and ROA spectra of vitamin B_12_ and its truncated analogs in the 900 to 350 cm^−1^ spectral region.

Experimental RR and RROA spectra of (CN)Cbl along with its truncated analogs are presented in Figure 3. Contrary to the UV-Vis spectra, Raman spectra of all three incomplete vitamin B_12_ derivatives were almost the same, particularly between 1650 and 1150 cm^−1^, where the most intense bands are located. Undoubtedly, Raman spectra of (CN)_2_Cbi and (CN)_2_Cbi-P species were identical in the whole spectral range, similarly to their UV-Vis and ECD spectra, because their structures differ only at distant places from the chromophore. However, in the case of the third analog, (CN)(H_2_O)Cby(OMe)_7_ (Figure 1), one can find a few modest differences in the Raman profile, especially below 900 cm^−1^ (Figure 4), that are related mostly to the replacement of the axial ligand CN by H_2_O. In general, consistent with the previous reports [32,41], RR spectra of cobalamins equipped with different axial ligands were strikingly similar, but at the same time, their electronic spectra (UV-Vis and ECD) differed significantly.

The first striking feature of the RR spectrum of (CN)(H_2_O)Cby(OMe)_7_ was a low-intensity band, centered at 1111 cm^−1^, that was less resolved for the other analogs and was present on their spectra as a shoulder (Figure 3). According to our recent study of cobalamins [41], that band is most likely involved in the corrin ring C-N stretching motions, but taking into consideration an additional seven -OMe groups in the (CN)(H_2_O)Cby(OMe)_7_ structure, one can say that the spectral feature may also be assigned to the O-CH_3_ vibrational stretching mode of CO_2_Me groups. Other spectral changes were observed between 900 and 500 cm^−1^ (Figure 4). Two closely lying bands at ~900 and ~850 cm^−1^ related to the CH_3_ rocking and C-C stretching of the side chains had the same intensity for (CN)(H_2_O)Cby(OMe)_7_, while in the case of (CN)Cbl, (CN)_2_Cbi and its phosphate derivative, the intensity of the ~900 cm^−1^ band was twice as low as for the one at ~850 cm^−1^. In the 730 to 750 cm^−1^ region, which is connected mostly with the CH_3_, CH_2_ rocking and corrin ring torsions, two separated bands were present for both (CN)_2_Cbi and (CN)_2_CbiP species (732 cm^−1^, 747 cm^−1^), and one spectral feature centered at 750 cm^−1^ with a shoulder at 732 cm^−1^ for (CN)(H_2_O)Cby(OMe)_7_ (Table 1). Conversely, in the RR spectrum of (CN)Cbl, the band at 732 cm^−1^ was more prominent while 750 cm^−1^ was less intense and hidden as a shoulder. Moreover, the 691 cm^−1^ band was much more intense for (CN)(H_2_O)Cby(OMe)_7_ than for (CN)Cbl and was not present for the other analogs. Furthermore, the band located at ~637 cm^−1^ for (CN)Cbl, (CN)_2_Cbi and (CN)_2_Cbi-P downshifted to 620 cm^−1^ for (CN)(H_2_O)Cby(OMe)_7_ and the intensity of the 584 cm^−1^ band increased in the order (CN)Cbl < (CN)_2_Cbi, (CN)_2_Cbi-P < (CN)(H_2_O)Cby(OMe)_7_. Last but not least, a shift was observed for the low-frequency band associated with the Co-C [30] or generally Co-L [41] stretching vibrations, from 518 and 484 cm^−1^ for (CN)Cbl or 519 cm^−1^ for (CN)_2_Cbi and (CN)_2_Cbi-P to 528 cm^−1^ for (CN)(H_2_O)Cby(OMe)_7_, which was due to the change of the axial ligand from Dmb or CN^−^ to CN^−^ or H_2_O.

Most of the RR spectral variations of the truncated analogs of (CN)Cbl result from the replacement of the lower axial ligand or are a consequence of the corrin ring conformational changes. However, as already mentioned, there were no evident differences between RR spectra of (CN)_2_Cbi and (CN)_2_Cbi-P, and it is therefore impossible to distinguish these two compounds, neither by RR, nor by UV-Vis or ECD spectroscopies. If we compare the RR spectrum of (CN)Cbl with its truncated analogs, observed differences were even more pronounced, thus replacement of the bulky Dmb moiety by a smaller lower axial ligand, e.g., CN^−^, affects the corrin ring conformation to a higher extent. This is in accordance with the change in the intensity and position of the *ν*_SA_ band and two other corrin ring stretching modes located above 1500 cm^−1^. First, we observed a pronounced shift of bands from 1543 (*ν*_SA_), 1577, and 1602 cm^−1^ for (CN)Cbl, to 1549, 1581, and 1612 cm^−1^, respectively, for all truncated analogs (Figure 3). Another change can be found in the bands’ intensity, which for (CN)Cbl was more or less similar for all three vibrational modes, while for truncated analogs the 1612 cm^−1^ band was much more intense than for the remaining two.

Experimental RROA spectra of all measured corrinoids are mostly positive, with an addition of a few negative, low-intensity bands (Figure 3). Analogously with our previous study in which we investigated cobalamins possessing a variously modified upper axial position [41], RROA spectra of truncated analogs of vitamin B_12_ are also bisignate, as a result of the excitation (532 nm) involving several closely-lying electronic states, with both negative and positive ECD. Positive RROA bands were due to the strong resonance upon the corrin-based π→π* transitions, characterized by negative ECD, that were located at 576 and 489 nm for (CN)_2_Cbi and (CN)_2_Cbi-P, and at 489 nm for (CN)(H_2_O)Cby(OMe)_7_. Presumably, negative RROA bands were most likely the result of the resonance upon the positive ECD electronic transition, located at 425 nm for (CN)_2_Cbi and (CN)_2_Cbi-P, and at 431 nm for (CN)(H_2_O)Cby(OMe)_7_ (Figure 2). ECD/UV-Vis (*g*-factor) ratios of the resonant electronic transitions and RROA/RR (CIDs) ratios, as expected for most cases, did not obey the SES relation i.e., CID = −1/2 *g*-factor, due to the resonance via multiple electronic states (Supplementary Materials, Tables S1 and S2).

In contrast to RR spectroscopy, which provided spectra where no changes could be found between (CN)_2_Cbi and (CN)_2_Cbi-P analogs, and only minor changes between (CN)_2_Cbi and (CN)(H_2_O)Cby(OMe)_7_ species were present, the RROA spectroscopy revealed to be superior. Similarly, as for the RR, RROA spectra of all truncated analogs were comparable in the corrin ring stretching region (1650 to 1450 cm^−1^), while the (CN)Cbl spectrum was noticeably different. However, unlike the position of the positive RROA *ν*_SA_ mode (~1548 cm^−1^), which was the same for all truncated analogs, the corrin ring motion had altered position and intensity for the studied analogs. We can clearly see that the intensity ratio of the 1548 and 1590 cm^−1^ bands for (CN)_2_Cbi was much higher than the corresponding intensity ratio of 1549/1583 cm^−1^ for (CN)_2_Cbi-P. What is more, negative 1612 cm^−1^ corrin ring stretching motion for (CN)_2_Cbi was split into two modes, both for the (CN)_2_Cbi-P (1612, 1606 cm^−1^) and (CN)(H_2_O)Cby(OMe)_7_ (1611, 1599 cm^−1^). Contrary to the RR spectra, some subtle differences were also found in the CH_3_ and CH_2_ bending vibrations region (1400 to 1300 cm^−1^) (Table 1). For (CN)_2_Cbi and (CN)(H_2_O)Cby(OMe)_7_, one can see two distinct modes at 1376 and 1357 cm^−1^ for the first one, as well as 1374 and 1355 cm^−1^ for the second one, while the (CN)_2_Cbi-P possess only one mode at 1371 cm^−1^. RROA spectra of (CN)_2_Cbi and (CN)_2_Cbi-P contained a band located at 1328/1321 cm^−1^, which was not present in the spectrum of (CN)(H_2_O)Cby(OMe)_7_. The most distinctive was the 1300 to 1100 cm^−1^ spectral region, where for the (CN)_2_Cbi, two separated, similarly intense, positive bands at 1230 and 1205 cm^−1^ could be found, while for (CN)_2_Cbi-P only one of higher intensity than for (CN)_2_Cbi was present (1202 cm^−1^). Moreover, two bands were present (1228 cm^−1^, 1203 cm^−1^) for the (CN)(H_2_O)Cby(OMe)_7_ but the 1203 cm^−1^ was higher in intensity. The 1230 cm^−1^ band was related mostly to CH_2_ wagging, and CH bending modes, while 1205 cm^−1^ to corrin ring C-C stretching and CH_2_ twisting [41]. This means that the presence of a phosphate group caused the structural changes around the side chains containing CH_2_ groups, as well as affecting the conformation of the corrin ring. A low wavenumber region in the RROA spectra is also distinctive for all incomplete analogs of vitamin B_12_, however, due to the low intensity of registered bands and low signal to noise ratio (S/N), the interpretation was not as straightforward as for other spectral regions (Figure 4). The major difference between truncated analogs, was a much higher intensity 432 cm^−1^ positive band for (CN)_2_Cbi compared to (CN)_2_Cbi-P and (CN)(H_2_O)Cby(OMe)_7_, that was ascribed to Co-C≡N bending, C≡N twisting modes and also to corrin ring torsion and bending vibrational modes. Between 900 and 600 cm^−1^, RROA spectra of (CN)_2_Cbi and (CN)_2_Cbi-P were almost the same and, similarly to the RR, the RROA spectrum of (CN)(H_2_O)Cby(OMe)_7_ had more pronounced differences compared to the remaining truncated analogs.

Surprisingly, noticeable changes in the resonance condition (a shift of the α/β electronic absorption bands with respect to the ROA 532 nm excitation line) of truncated vitamins compared to the parent vitamin B_12_, did not change the overall RR and RROA profiles of the studied compounds. However, slight, but not negligible, differences could be found in the case of intensity ratios of RROA/RR (CID values) of the corresponding bands. What we have observed is that for (CN)_2_Cbi and (CN)_2_CbiP, where the α/β electronic bands showed the bathochromic shift compared to the (CN)Cbl spectra, the CID ratios of *ν*_LA_ (1501 cm^−1^) were more than two times smaller than for the (CN)Cbl. (CN)_2_Cbi and (CN)_2_CbiP *ν*_LA_ CID ratios equal 2.0 × 10^−4^ and 1.6 × 10^−4^, respectively, while for the (CN)Cbl it was 4.0 × 10^−4^ (Table S2). In the case of (CN)(H_2_O)Cby(OMe)_7_, where the α/β electronic bands showed the hypsochromic shift, the CID ratio of *ν*_LA_ was higher than that for the remaining truncated analogs (2.5 × 10^−4^), but still lower than that for (CN)Cbl. This means that, in comparison to their parent RR spectra, RROA spectra of (CN)_2_Cbi and (CN)_2_CbiP were less resonantly enhanced than was observed for (CN)(H_2_O)Cby(OMe)_7_ and especially for the (CN)Cbl. It was also clearly visible in Figure 3, where the RR intensity of (CN)Cbl and incomplete analogs were comparable, while in the case of RROA, (CN)_2_Cbi, and (CN)_2_CbiP spectra, they possessed much lower intensity compared to the (CN)(H_2_O)Cby(OMe)_7_ and (CN)Cbl. Therefore, changing the resonance conditions did not affect RR and RROA profiles significantly but alters the RROA/RR intensity ratios.

## 3. Materials and Methods 

### 3.1. Sample Preparation

Cyanocobalamin ((CN)Cbl) was purchased from Sigma-Aldrich (Saint Louis, MO, USA). Truncated vitamin B_12_ derivatives: dicyanocobinamide ((CN)_2_Cbi), dicyanocobinamide phosphate ((CN)_2_Cbi-P), and cyanoaqua cobyrinic acid heptamethylester ((CN)(H_2_O)Cby(OMe)_7_) were synthesized according to reported procedures [16,18].

### 3.2. Spectroscopic Measurements (UV-Vis/ECD, Raman/ROA)

Raman and ROA spectra of vitamin B_12_ and truncated vitamin B_12_ derivatives dissolved in distilled water (*c* = 0.1 mg/mL) were registered using a ChiralRAMAN-2X spectrometer (BioTools Inc., Jupiter, FL, USA) at a resolution of 7 cm^−1^ in the range of 2500 to 250 cm^−1^ employing the excitation wavelength of 532 nm. The spectra were measured in ROA quartz optical cells with anti-reflective coating. The laser power of 200 mW, integration time of 4 s and 24 h (for (CN)Cbl) or 60 h (for (CN)_2_Cbi, (CN)_2_Cbi-P and (CN)(H_2_O)Cby(OMe)_7_) acquisition time was used for Raman and ROA measurements of studied compounds. For truncated cobalamin derivatives the longer accumulation time was used (60 h), due to the insufficient S/N ratio after 24 h acquisition. Due to the limited stability of truncated cobalamin derivatives under laser irradiation (over 12 h), the final 60 h spectra were averaged over five 12 h measurements (after rejection cosmic ray artifacts and an incorrect ROA compensation) of freshly prepared solutions. All solutions were passed through the Millex^®^ (Merck Millipore^TM^, Burlington, MA, USA) syringe PTFE filters (pore size 0.2 μm) to remove solid impurities before running the measurement. Raman spectra were corrected by subtracting the spectrum obtained for the solvent using OPUS software (OPUS 7.2, Bruker Optik GmbH, Ettlingen, Germany). The baseline of Raman and ROA spectra were subtracted by asymmetric least squares smoothing method, and then smoothed with the ten-point Savitzky–Golay procedure using OriginPro software (OriginPro2018, OriginLab Corporation, Northampton, MA, USA).

UV-Vis and ECD spectra were recorded in the 220 to 700 nm spectral range using quartz cells with a path length of 10 mm, a scanning speed of 100 nm/min, a bandwidth of 1 nm, 0.5 s response time, and an accumulation of a single scan on the Jasco J-815 spectrometer. The concentration of all samples was 0.1 mg/mL. The baseline of the registered spectra was corrected using JASCO software (Jascow32, JASCO Corporation, Easton, MD, USA).

## 4. Conclusions

While it is not possible to distinguish between (CN)_2_Cbi and (CN)_2_CbiP by means of RR, UV-Vis, or ECD spectroscopy, RROA turned out to be a sensitive probe for subtle structural alterations in the molecules measured in resonance with multiple electronic states. In the presented study, not only the RR spectra of two analogs, i.e., (CN)_2_Cbi and (CN)_2_CbiP, were virtually the same, but also their UV-Vis and ECD spectra, which means that only the RROA method allows for successful distinction between these, only slightly structurally different, compounds. What is even more remarkable, the only structural difference between both molecules is the phosphate group located in the *f*-side chain, which is quite distant from the corrin ring, and obviously, that substitution should affect the corrin macrocycle conformation very slightly, but still enough to be detected on the respective RROA spectra. ROA spectroscopy is therefore highly sensitive to subtle alteration in a structure of truncated vitamins, but the ECD method still shows spectral changes accompanying axial ligands incorporation to the central cobalt ion, e.g., (CN)Cbl vs. (CN)(H_2_O)Cby(OMe)_7_. The current study is an important step in the dissemination of using resonantly enhanced ROA spectroscopy in studying the complex structure of corrinoids, which may prove crucial for a better understanding of their biological role.

## Figures and Tables

**Table 1:** Assignments of the most characteristic Raman and ROA bands of vitamin B_12_ ((CN)Cbl) [41].

Raman/cm^−1^	ROA/cm^−1^	Assignments
1602	-	C=C str (14-15), C=N str (16-24)
1577	1578 (+)	C=C str (5-6; 10-11), C-H bend (10), C=N str (16-24; 4-21)
1543	1546 (+)	C=C str (5-6; 10-11; 14-15), C=N str (9-22; 11-23; 16-24; 4-21)
1501	1501 (+)	C=C str (5-6; 9-10; 14-15), C=N str (4-21; 11-23; 16-24; B2-B1; B2-B3), C-H bend (10; B2), CH_3_ bend asym, CH_2_ bend (scissoring)
1350	1344 (+)	CH_2_ wagg, CH bend
1228	1228 (+)	CH_2_ wagg, CH bend, NH bend, CH_3_ rock
1205	1205 (+)	C-C str (corrin ring), CH_2_ twist, CH bend, CH_3_ rock
1168	1168 (+)	C-N str (6-22; 14-23), C-C str (corrin ring), CH_2_ twist, CH bend, CH_3_ rock
1105s	1109 (−)	C-N str (6-22; 14-23), NH_2_ rock, CH_2_ twist, CH bend
890	895s (+) 877 (+)	CH_3_ and CH_2_ rock, C-C str (7-37; 37-38; 8-9;6-7; 8-41; 9-10; 12-46), Co-N str (22; 23), C-C str (1-2; 1-19; 1-20; 3-4; 4-5; 5-35; 6-7; 7-8; 7-36; 31-32), C-N str (1-21)
732	732 (+)	CH_3_ and CH_2_ rock, corrin ring tors and bend, NH_2_ twist, CCC bend, CC, CN tors, benzimidazole C-C str
696	695 (−)	CH_2_ rock, NH_2_ twist, corrin ring tors and bend, CCC bend, CC, CN tors
636	635 (+)	NH_2_ twist, corrin ring tors and bend
584	585 (+)	NH_2_ twist
518	518 (+)	corrin ring tors and bend, NH_2_ twist, CCC bend, CC, CN tors
496	497 (−)	Co-C≡N bend, C≡N twist, corrin ring tors and bend, benzimidazole ring breathing, Co-N bend (B3), O-H bend (7R)
427	429 (+)	Co-C≡N bend, corrin ring tors and bend, CCN, CNC bend, CC, CN tors, C≡N twist, O-H bend (7R)

Abbreviations: str—stretching; bend—bending; sym—symmetric; asym—asymmetric; wagg—wagging; twist—twisting; rock—rocking; tors—torsion; s—shoulder, (+) and (–)—positive and negative ROA intensities, numbers in brackets denote atoms involved in vibrations accordingly to the Figure 1 numeration.

## Supplementary Materials

The following are available online, Figure S1: UV-Vis and ECD spectra of vitamin B_12_ and its truncated analogs measured before and after RROA experiments involving ROA laser exposure. All samples (*c* = 0.1 mg/mL) were measured in cells with a path length of 1 mm and an accumulation of five scans; Table S1: Dissymmetry factor (*g*-factor, ECD/UV-Vis) values, plotted for selected transitions, obtained from (CN)Cbl, (CN)_2_Cbi, (CN)_2_Cbi-P, and (CN)(H_2_O)Cby(OMe)_7_ experimental spectra; Table S2: CID (ROA/Raman) values, plotted for selected transitions, obtained from (CN)Cbl, (CN)_2_Cbi, (CN)_2_Cbi-P, and (CN)(H_2_O)Cby(OMe)_7_ experimental spectra.
