# Supplementary material for: Resonance Raman Optical Activity Spectroscopy in Probing Structural Changes Invisible to Circular Dichroism Spectroscopy: A Study on Truncated Vitamin B12 Derivatives
Source: Molecules. 2020 Sep 24;25(19):4386. doi: 10.3390/molecules25194386 (PMC7584048; doi:10.3390/molecules25194386)
Supplement: Supplementary file 1 [file molecules-25-04386-s001.docx]

*Supplementary Material*

Resonance Raman optical activity spectroscopy in probing structural changes invisible to circular dichroism spectroscopy: a study on truncated vitamin B_12_ derivatives

Ewa Machalska ^1,2^, Grzegorz Zajac ^2^, Monika Halat ^1,2^, Aleksandra J. Wierzba ^3^, Dorota Gryko ^3,^* and Malgorzata Baranska ^1,2,^*

^1^ Faculty of Chemistry, Jagiellonian University, Gronostajowa 2, Krakow 30-387, Poland; ewa.machalska@doctoral.uj.edu.pl (E.M.); monika.halat@doctoral.uj.edu.pl (M.H.)

^2^ Jagiellonian Centre for Experimental Therapeutics (JCET), Jagiellonian University, Bobrzynskiego 14, Krakow 30-348, Poland; zajac@chemia.uj.edu.pl (G.Z.)

^3^ Institute of Organic Chemistry, Polish Academy of Sciences, Kasprzaka 44/52, Warsaw 01-224, Poland; aleksandra.wierzba@icho.edu.pl (A.J.W.); dorota.gryko@icho.edu.pl (D.G.)

***** Correspondence: m.baranska@uj.edu.pl (M.B.); dorota.gryko@icho.edu.pl (D.G.)





**Figure S1**. UV-Vis and ECD spectra of vitamin B_12_ and its truncated analogs measured before and after RROA experiments involving ROA laser exposure. All samples (c=0.1 mg/mL) were measured in cells with a path length of 1 mm and an accumulation of 5 scans.

**Table S1**. Dissymmetry factor (*g-*factor, ECD/UV-Vis) values, plotted for selected transitions, obtained from (CN)Cbl, (CN)_2_Cbi, (CN)_2_Cbi-P and (CN)(H_2_O)Cby(OMe)_7_ experimental spectra.

| (CN)Cbl | | (CN)_2_Cbi | | (CN)_2_Cbi-P | | (CN)(H_2_O)Cby(OMe)_7_ | |
| --- | --- | --- | --- | --- | --- | --- | --- |
| *λ / nm* | *g* / 10^-4^ | *λ / nm* | *g* / 10^-4^ | *λ / nm* | *g* / 10^-4^ | *λ / nm* | *g* / 10^-4^ |
| 544 | -2.4 | 576 | -0.9 | 576 | -0.9 | 552 | 6.2 |
| 507 | -0.5 | 536 | -0.7 | 536 | -1.1 | 526 | -1.0 |
| 521 | -2.3 | 489 | -5.8 | 489 | -5.2 | 496 | -7.2 |
| 483 | -8.5 | 425 | 62 | 425 | 58 | 489 | -8.2 |
| 433 | 50 | 416 | 48 | 416 | 47 | 431 | 41 |
|  |  |  |  |  |  | 404 | 43 |

**Table S2**. CID (ROA/Raman) values, plotted for selected transitions, obtained from (CN)Cbl, (CN)_2_Cbi, (CN)_2_Cbi-P and (CN)(H_2_O)Cby(OMe)_7_ experimental spectra.

| (CN)Cbl | | (CN)_2_Cbi | | (CN)_2_Cbi-P | | (CN)(H_2_O)Cby(OMe)_7_ | |
| --- | --- | --- | --- | --- | --- | --- | --- |
| *v / cm^-1^* | *CID*/ 10^-4^ | *v / cm^-1^* | *CID*/ 10^-4^ | *v / cm^-1^* | *CID* / 10^-4^ | *v / cm^-1^* | *CID* / 10^-4^ |
| - | - | 1612 | -0.5 | 1612 | -0.5 | 1612 | -0.9 |
| - | - | - | - | 1606 | -0.3 | 1599 | -0.6 |
| - | - | 1590 | 1.5 | 1583 | 2.9 | 1586 | 1.3 |
| 1578 | 4.1 | 1581 | -1.6 | 1581 | 1.8 | 1581 | 1.0 |
| 1546 |  | 1548 | 7.9 | 1549 | 10 | 1548 | 7.0 |
| 1501 | 4.3 | 1501 | 2.0 | 1501 | 1.6 | 1501 | 2.5 |
| 1398 | 1.8 | 1399 | 6.2 | 1399 | 6.2 | 1399 | 7.4 |
| 1376 | 1.9 | 1376 | 4.6 | 1371 | 3.5 | 1374 | 5.2 |
| - | - | 1357 | 3.3 | 1350 | 1.7 | 1356 | 3.4 |
| 1310 | -3.3 | 1328 | 5.8 | 1321 | 3.6 | - | - |
| 1228 | 4.2 | 1230 | 1.6 | 1229 | 0.3 | 1228 | 2.2 |
| 1205 | 3.6 | 1205 | 2.8 | 1202 | 0.5 | 1203 | 1.2 |
| 1168 | 2.4 | 1161 | 1.0 | 1157 | 1.3 | 1161 | 2.3 |
| 1144 | 1.0 | 1139 | -1.4 | 1137 | -1.7 | 1139 | 0.3 |
| 1109 | -1.4 | - | - | - | - | 1113 | 3.8 |
| 776 | -4.0 | 760 | 4.6 | 758 | 4.6 | 748 | 3.3 |
| 732 | 2.2 | 726 | 3.5 | 714 | 3.0 | 732 | 5.1 |
| 636 | 5.7 | 634 | 2.3 | 637 | 2.2 | 621 | 1.8 |
| 518 | 8.8 | 519 | 6.8 | 518 | 7.1 | 524 | 3.4 |
| 497 | -40 | 493 | -22 | - | - | 489 | -10 |
| 427 | 6.1 | 432 | 4.4 | 431 | 0.9 | 430 | 1.8 |
| 406 | 3.7 | 422 | 0.8 | - | - | 414 | 2.3 |
